# COVID-19 vaccines: awareness, attitude and acceptance among undergraduate University students

**DOI:** 10.1186/s40545-021-00397-6

**Published:** 2022-04-26

**Authors:** Firew Asres, Belachew Umeta

**Affiliations:** 1 Department of Pharmaceutical Sciences, School of Pharmacy, Jimma University, Jimma, Oromia Ethiopia; 2 School of Pharmacy, Institute of Health, Jimma University, Jimma, Oromia Ethiopia

**Keywords:** COVID-19, Vaccine, Novel coronavirus vaccine, Jimma

## Abstract

**Background:**

The myths and conspiracy theories on the COVID-19 vaccine cause people to be hesitant and maleficent towards the vaccine.

**Objectives:**

To assess COVID-19 vaccine-related awareness, attitude and acceptance and to assess reasons for refusing the vaccine among undergraduate Jimma University Institute of Health students.

**Methods:**

A cross-sectional study was conducted among 387 undergraduate students of Jimma University Institute of Health. Self-administered questionnaires were used to collect the data and summarized by descriptive statistics. A multivariable regression model was used to assess predictable variables for good awareness and positive attitude. A *p* value of < 0.05 was used to declare the statistical association.

**Results:**

Only 41% of the students had a good awareness of the COVID-19 vaccine, and more than half, 224 (57.9%) of them had a positive attitude towards the COVID-19 vaccine. Age [(AOR: 95% CI) 1.18 (1.03, 1.35)] and having good awareness [(AOR: 95% CI) 2.39 (1.55, 3.68)] were associated with positive attitude of students towards the COVID-19 vaccine. However, only 27.1% of the students were willing to take the vaccine for COVID-19. Afraid of long term effects (49.1%), not being convinced of the safety standards (38.8%), lack of information about the vaccine (37.2%), and too short time for development (39.9%) was common reasons for refusing the COVID-19 vaccine.

**Conclusions:**

According to the present study, the majority of the participants had a positive attitude towards COVID-19 vaccine. However, only less than half of the participants had a good awareness of the vaccine. In addition, the acceptability of the vaccine is low. Afraid of long term effects, not being convinced of the safety profile, lack of information about the vaccine, and the time used for the development were the common reasons for refusing the vaccine. Therefore, all stakeholders are advised to increase awareness, positive attitude, and acceptance of the vaccine.

## Background

Coronavirus disease 2019 (COVID-19) is caused by severe acute respiratory syndrome coronavirus-2 (SARS-CoV-2) [1]. The first case of COVID-19 was reported from China Wuhan city on December 12, 2019 [2]. The virus is transmitted through large droplets generated during coughing or sneezing of symptomatic and asymptomatic patients [3]. During the outbreak, the only strategy was to reduce the spread. Wearing masks, alcohol-based hand sanitizer, social distancing, travel restrictions, school closures, and partial or complete lockdowns were used as the prevention strategy [4]. So far, they were able to slow the disease progression.

On March 11, 2020, the World Health Organization declared the outbreak a pandemic [5]. Since its emergency, the pandemic has spread rapidly, resulting in a global health, social, economic, and political crisis [6]. Political scientists cannot eradicate the virus or cure the disease. However, the impact of COVID-19 will ultimately be determined by politics. Currently, there is well-developed scientific knowledge of crisis policy in specific areas, such as finance, energy, natural disasters and pandemics [7]. The interpretation and response of a pandemic have always been a political act as the decision to impose border control, quarantine the population, public information management, and take an attitude towards others is never freed from such things. Political and medical leaders of governments, national agencies, and international organizations, such as the World Health Organization (WHO) manage data and figures to control the pandemic [8].

The virus has infected people in over 220 countries. More than 213,345,924 people were infected, and 4,454,131 deaths were reported on August 24, 2021.

Myths and conspiracies about the vaccination are proliferating, putting the community in a bind. Myths and conspiracies may create apprehension and malice toward the COVID-19 vaccination. Vaccine hesitancy was named one of the top ten worldwide dangers to public health by the World Health Organization [8, 9]. Three factors influence vaccine acceptance. These are confidence, convenience, and complacency [10]. Confidence is trust in the safety, efficacy, delivery system, and policymakers [11]. Many people doubt safety, and it is challenging for healthcare providers, policymakers, community leaders, and governments [12–14]. The relative ease of availability to the vaccine is vaccination convenience [15]. Vaccine complacency is a low recognized risk of vaccine-preventable illness and unfavorable attitudes about vaccines.

AP-NORC poll in the United States reported only 50% of Americans were willing to take the COVID-19 vaccination, 31% were unsure, and 20% refused the vaccine [16]. Another study found that around 58% were willing to take the vaccination, 32% were unsure, and 11% did not intend to be vaccinated [17]. The other study reported 67% of Americans were willing to accept the vaccine [18]. The study in selected Universities of Northeast Ethiopia revealed 69.3% of the participants were intended to be vaccinated as soon as the vaccine was available [19]. The other study conducted in China reported that 91.3% of the participants accepted the vaccine [20].

The COVID-19 Vaccines Global Access (COVAX) facility strives to deliver a minimum of 2 billion doses of the vaccine to concerned countries of the world in 2021, which includes at least 1.3 billion doses funded by donors to the 92 lower income countries. COVAX facility allocated 7,620,000 doses of COVID-19 vaccine for Ethiopia and about 2,184,000 doses imported by the time the study was conducted.

Ethiopia started vaccinations on March 20 and has only immunized a fraction of the population. As the Ministry of Health plans, 20% of the population in Ethiopia will be vaccinated by the end of 2021. However, it is critical to comprehend the awareness, attitude, and willingness to accept the vaccine. Therefore, assessing awareness, attitude, acceptance and the reasons for refusal will aid in developing and implementing an effective pandemic-control strategy [1]. Therefore, the study aimed to assess Jimma University undergraduate institute of health students awareness, attitudes, acceptance, and the reasons for refusing the vaccine.

## Methods

### Setting and period

The study was conducted at Jimma University. Jimma University is located in Jimma city 350 km Southwest of Addis Ababa, the capital city of Ethiopia. Jimma University is one of the largest and comprehensive public research Universities in Ethiopia. Jimma University Institute of Health has three faculties. These are Health Sciences, Medical science, and Public Health.

### Study design

A cross-sectional study design was employed.

### Sample size and sampling technique

The sample size was determined using the single population proportion formula: *n* = *Z*^2^*P*(1−*P*)/*d*^2^, where *P* = 0.5 (50%) and *Z* = 1.96 (95% CI), degree of error = 5% and non-respondent rate = 5%. Finally, after adding a 5% non-response rate (384 + 19), the total sample size was 403. Students were selected randomly.

### Study population

Randomly selected undergraduate students of Jimma University Institute of Health were included in the study. Students who were unwilling to participate and unable to give the required information were excluded from the study.

### Data collection tools and technique

A structured questionnaire was adopted from previous studies [17, 21]. The questionnaire was prepared in English. The questionnaire consists of the following parts.

Sociodemographic characteristics: age, sex, religion, department, year of study and area of origin.

Awareness towards COVID-19 vaccines: ever heard of about COVID-19 vaccine, where you get COVID-19 vaccine-related information, COVID-19 vaccines are effective, overdose of COVID-19 vaccines are dangerous, COVID-19 vaccines might cause allergic reaction and COVID-19 vaccines might increase autoimmune disease.

Attitude towards COVID-19 vaccines: I believe a vaccine can help control the spread of COVID-19, if I knew I had been infected with COVID-19 before, I will get the vaccine if it is available, when everyone else is vaccinated against COVID-19, then I do not have to get vaccinated, the COVID-19 vaccine is essential for us, the newly discovered COVID-19 vaccine is safe, I will take COVID-19 vaccine without any hesitation, I will also encourage my family/friends/relatives to get vaccinated, it is not possible to reduce the incidence of COVID-19 without vaccination, the COVID-19 vaccine should be distributed equally to all of us.

Willingness to take vaccine: do you take COVID-19 vaccine if freely available for students?

Reasons for refusing the vaccine: too short a time for development and testing, not convinced of the safety standards of vaccine, afraid of long-term effects, harmful substance in the vaccine, vaccine causing COVID-19, vaccine will not help, it is not effective, not safe with lower efficacy, I want to build my immunity/I prefer other ways of protection, do not trust system/ something that nobody knows about, vaccine not reliable, doubts about the vaccine due to short time for development, do not believe COVID-19 is a threat, COVID-19 is overrated, no vaccine is needed, It is biological weapon, I am not a guinea pig, I would not like to be like who first took it, do not have enough information about vaccine, political game, the vaccine is a money-making venture.

### Outcome variable measurement

The measurement of awareness and attitude was adopted from previous researches [19, 20, 23]. Six yes/no items were included as awareness assessment tools. The correct response was recorded as 1 and 0 for incorrect. The maximum score was 6, and the minimum score was 0. The Awareness was categorized as good or poor. Participants scoring mean or more were categorized as they have good awareness and vice versa.

The attitude section contains eight (8) yes/no items. The correct answer was recorded as 1, and 0 for the incorrect. The maximum score was 8, and the minimum was 0. In addition, the attitude was categorized as a positive and negative attitude similarly to awareness classification.

### Data processing and analysis

The data were entered and cleaned using Epi Info 3.1 software and exported to SPSS version 26 for further analysis. Descriptive statistics were used for describing and summarizing the data and chi-square was used to pin out the association of the dependent variable with the independent variables. A multivariable logistic regression model was performed to determine factors associated with the awareness and attitude of participants towards the COVID-19 vaccine. A variable with a *p* value of less than 0.25 in binary logistic regression was eligible for multivariable logistic regression. *p* value < 0.05 was used to declare the significant association.

### Ethical considerations

A permission letter was obtained from the School of Pharmacy, Jimma University. The work was done as the duty of workers to advise students. Verbal consent was obtained from the participants after the purpose and methods of the study had been explained in detail. All of their responses were kept confidential and anonymous.

## Results

### Sociodemographic characteristics of participants

The response rate of the study was 96.03% (387/403). More than half, 252 (65.1%) of the participants were males, and the age (mean ± SD) was 21.97 ± 1.67. Around 39% of the participants were followers of the Orthodox religion. 108 (27.9%) of the participants were medicine students, and 41.1% were 2nd year students. The majority, 72.4% of them comes from urban parts of the country (Table 1).

**Table 1:** Sociodemographic characteristics of participants

Characteristics	Frequency (%)	
Age (mean ± SD)		21.97 ± 1.63
Sex		
Male		252 (65.1)
Female		135 (34.9)
Religion		
Muslims		81 (20.9)
Orthodox		151 (39.0)
Protestant		132 (34.1)
Others		23 (5.9)
Department		
Medicine		108 (27.9)
Medical laboratory		81 (20.9)
Pharmacy		70 (18.1)
Health officer		39 (10.1)
Nursing		31 (8.0)
Anesthesia		29 (7.5)
Environmental health		20 (5.2)
Midwifery		9 (2.3)
Year of study		
2nd		159 (41.1)
3rd		89 (23.0)
4th		102 (26.4)
5th		26 (6.7)
6th		11 (2.8)
Residency		
Urban		280 (72.4)
Rural		107 (27.6)

### Awareness of students about COVID-19 vaccine

The awareness score (mean ± SD) of the participants was 3.34 ± 1.30. One hundred and fifty-nine, 159 (41.1%) of participants had a good awareness of COVID-19 vaccines. Chi-square performed indicated the association of year of study with awareness towards the COVID-19 vaccine (Table 2).

**Table 2:** COVID-19 vaccine awareness among Jimma University Institute of Health students

Variables	Awareness status		Sig
	Good	Poor	
Sex			
Male	98	154	0.230
Female	61	74	
Department			
Medicine	43	65	0.481
Medical laboratory	30	51	
Pharmacy	33	37	
Health officer	16	23	
Nursing	13	18	
Anesthesia	13	16	
Environmental health	5	15	
Midwifery	6	13	
Year of study			
2nd	55	104	0.002
3rd	28	61	
4th	57	45	
5th	14	12	
6th	5	6	
Place of residency			
Urban	120	160	0.252
Rural	39	68	
Religion			
Muslims	36	45	0.447
Orthodox	67	84	
Protestant	48	84	
Others	8	15	

### Attitude of students towards COVID-19 vaccine

The attitude score (mean ± SD) of the participants was 4.02 ± 2.47. Two hundred and twenty-four, 224 (57.9%) of the participants have a positive attitude towards the COVID-19 vaccines (Table 3).

**Table 3:** Attitude of participants towards the COVID-19 vaccine

Variables	Attitude status		Sig
	Positive	Negative	
Sex			
Male	147	105	0.806
Female	77	58	
Department			
Medicine	63	45	
Medical laboratory	54	27	
Pharmacy	32	38	
Health officer	20	19	0.196
Nursing	18	13	
Anesthesia	19	10	
Environmental health	11	9	
Midwifery	7	2	
Year of study			
2nd	83	76	
3rd	51	38	0.157
4th	68	34	
5th	14	12	
6th	8	3	
Place of residency			
Urban	161	119	0.806
Rural	63	44	
Religion			
Muslims	51	30	
Orthodox	86	65	0.60
Protestant	76	56	
Others	11	12	

### COVID-19 vaccine acceptance among participants

**Fig. 1:**
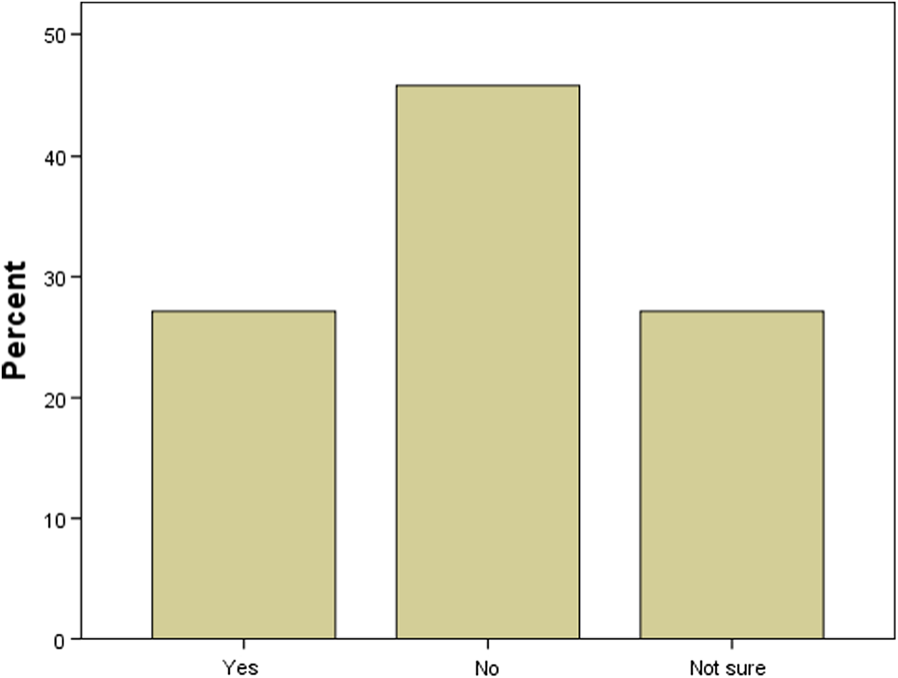
COVID-19 Vaccine acceptance among participants

Only 27.1% of participants have expressed their willingness to take the COVID-19 vaccine, and 45.7% of students were unwilling to have the COVID-19 vaccine (Fig. 1).

### Reasons for refusing COVID-19 vaccine

The participants were listed different factors for refusing to take the COVID-19 vaccine. From these: afraid of long-term effects, not convinced of the safety standards of vaccine, do not have enough information about the vaccine and too short time for development were some of them (Table 3).

### Factors associated with awareness and attitude of students towards the COVID-19 vaccine

No significant difference was observed among Jimma University Institute of Health students regarding their awareness of COVID-19 vaccines. However, there is a relationship between the age of students and attitude towards the COVID-19 vaccines (95% CI 1.03, 1.35) (Table 4).

**Table 4:** Factors associated with knowledge and attitude of participants towards COVID-19 vaccine

Sociodemographic characteristics	Awareness		AOR (95% CI)	Attitude COR (95% CI)		AOR (95% CI)
	COR (95% CI)	p-value		COR (95% CI)	p-value	
Sex						
Male	1		1	1.05 (0.69, 1.61)	0.806	
Female	1.30 (0.85, 1.98)	0.231	1.39 (0.90, 2.14)	1		
Age	1.11 (0.98, 1.26)	0.099	1.10 (0.96, 1.27)	1.20 (1.05, 1.37)	0.007	1.18 (1.03, 1.35)*
Department						
Health sciences^a^	1			1		
others^b^	0.93 (0.59, 1.46)	0.752		1.03 (0.65, 1.61)	0.911	
Year of study						
1–4	1		1	1		
> 4	1.58 (0.80, 3.12)	0.185	1.35 (0.64, 2.83)	1.08 (0.54, 2.14)	0.838	
Religion						
Christians	1			1		
Muslims	1.19 (0.73, 1.95)	0.49		1.31 (0.79, 2.16)	0.298	
Place of residency						
Urban	1.31 (0.83, 2.07)	0.252		1		
Rural	1			1.06 (0.67, 1.66)	0.806	
Awareness status						
Good				2.47 (1.61, 3.79)	< 0.001	2.39 (1.55, 3.68)*
Poor				1		1

^a^Students of Pharmacy, medical laboratory, nursing, anesthesia, environmental health ^b^Dentistry, health officer and medicine students. *significance

## Discussion

The availability and efficacy of the COVID-19 vaccine are vital to control the pandemic. Policymakers and health authorities must ensure acceptance and trust from the community and healthcare workers, because hesitation and delay may result in vaccination refusal. These could lead to devastating effects on public health and hinder the healthcare system’s ability to accommodate the challenges of the pandemic. Community awareness, attitude and acceptance affect public health and the healthcare system to withstand the challenges of the pandemic [22]. Vaccination is an effective way of controlling infectious diseases. However, the success is challenged by individuals and groups who choose to delay or refuse vaccines. Vaccine hesitancy is one cause for decreasing vaccine coverage and increasing the risk of vaccine-preventable disease outbreaks and epidemics [23].

According to the present study, only less than half of participants had a good awareness of the COVID-19 vaccine. The result was comparable with the study conducted on the web in Ethiopia reporting 40.8% of the participants had a good awareness of the COVID-19 vaccine [25]. However, the finding was lower than the study conducted in Ethiopia on adult populations in which 74% of participants have good knowledge of the COVID-19 vaccine [26]. The discrepancy might be due to differences in the study population and sample size. However, statistical analysis showed no association of sociodemographic characteristics with their respective awareness of the COVID-19 vaccine.

More than half, 58% of participants had a positive attitude towards the COVID-19 vaccine. The finding was comparable with the studies conducted in Ethiopia on health professionals in which around 42.3% of the participants had a positive attitude [24]. However, the finding was higher than the e-based survey conducted in Ethiopia in which only 24.2% of the participants had a positive attitude towards the COVID-19 vaccine [25]. The discrepancy might be due to the method of data collection in which the previous study used web-based data collection and differences in the study unit. Statistically, age and having good awareness about the COVID-19 vaccine was associated with attitude, and the study conducted in Ethiopia also reported the association of age with the attitude towards the COVID-19 vaccine [24, 26].

The number of participants who expressed their willingness to take the COVID-19 vaccine was small. Only 27% of the participants were willing to take the vaccine. This figure was smaller than an institutional study conducted in Ethiopia, which reported that 61% of the participants had expressed their willingness to take vaccines whenever available [27]. The other study conducted in Ethiopia reported around 62.6% of COVID-19 vaccine acceptance [26]. The discrepancy might be due to sample size differences and differences in study participants.

The reasons for refusing the COVID-19 vaccine were: not convinced of the safety standards of the vaccine, Afraid of long-term effects, do not have enough information about the vaccine, too short time for development and Doubts about the vaccine due to short time of development were the most reported reasons (Tables 5).

**Table 5:** Participants reasons for refusing the COVID-19 vaccine

Reasons for refusing to take COVID-19 vaccine	Frequency (%)
To short time for development	131 (33.9)
Not convinced of the safety standards of vaccine	150 (38.8)
Afraid of long-term effects	191 (49.1)
Harmful substance in vaccine	104 (26.9)
Vaccine causing COVID-19	47 (12.1)
Vaccine will not help, it is not effective	46 (11.9)
Not safe with lower efficacy	57 (14.7)
I want to build my own immunity/I prefer other ways of protection	89 (23)
Do not trust system/something that nobody knows about	109 (28.2)
Vaccine not reliable	71 (18.3)
Doubts about the vaccine, due to short time for development	123 (31.8)
Do not believe COVID-19 is a threat	35 (9.0)
COVID-19 is overrated, no vaccine is needed	26 (6.7)
It is Biological weapon	68 (16.8)
I am not a guinea pig	47 (12.1)
I would not like to be like who first took it	38 (9.8)
Do not have enough information about vaccine	144 (37.2)
Political game	118 (30.5)
Vaccine is a money-making venture	49 (12.7

## Conclusions

According to the present study, the majority of participants had a positive attitude towards COVID-19 vaccine. However, only less than half of the participants had a good awareness of the vaccine. In addition, the acceptability of the vaccine is low. Afraid of long term effects, not being convinced of the safety profile, lack of information about the vaccine, and the time used for the development were the common reasons for refusing the vaccine. Therefore, all stakeholders are advised to increase awareness, positive attitude, and acceptance of the vaccine.

## Data Availability

The data set used for analysis is available from the corresponding author upon request.
